# Convective Drying of Avocado Seeds: Mass Transfer Thermodynamics and Multi-Response Optimization of Functional and Phytochemical Properties

**DOI:** 10.3390/foods15142438

**Published:** 2026-07-09

**Authors:** Mayra Deyanira Ramírez-Aguirre, Ricardo de Jesús Montiel-López, Tomás García-Cayuela, Viridiana Tejada-Ortigoza, Veronica Rodriguez-Martinez, Luis Eduardo Garcia-Amezquita

**Affiliations:** 1 Escuela de Ingeniería y Ciencias, Tecnologico de Monterrey, Ave. General Ramón Corona 2514, Zapopan 45138, Jalisco, Mexico; a01114369@tec.mx (M.D.R.-A.); tomasgc@tec.mx (T.G.-C.); veronica.rodriguezmz@tec.mx (V.R.-M.); 2 Escuela de Ingeniería y Ciencias, Tecnologico de Monterrey, Ave. Eugenio Garza Sada 2501, Monterrey 64700, Nuevo Leon, Mexico; a01740943@tec.mx (R.d.J.M.-L.); viri.tejada@tec.mx (V.T.-O.)

**Keywords:** avocado seed by-product, convective drying kinetics, mass transfer thermodynamics, dietary fiber functionality, phytochemical preservation, multi-response optimization

## Abstract

Avocado seeds represent an underutilized agro-industrial by-product rich in dietary fiber and bioactive compounds. This study evaluated the impact of convective drying (45–75 °C, 3–9 mm thickness, 0.5–2.5 m s^−1^ air velocity) on the mass transfer kinetics, techno-functional properties, and phytochemical stability of the seed matrix. The Midilli model accurately described dehydration kinetics, with effective diffusivities around 10^−9^ m^2^ s^−1^. Principal Component Analysis of the evaluated parameters revealed trade-offs between drying efficiency and phytochemical preservation. While the lignocellulosic fiber matrix remained relatively stable, preserving its hydration and oil retention capacities independently of thermal severity, prolonged processing times resulted in lower phenolic acid content and promoted non-enzymatic browning. Crucially, high air velocities were associated with higher retention of thermolabile bioactives, potentially due to accelerated moisture removal and shorter cumulative thermal exposure. A multi-response desirability approach established three optimized processing scenarios, yielding a phytochemical-rich concentrate (45 °C, 3 mm, 2.5 m s^−1^), a highly soluble ingredient (75 °C, 8.7 mm, 0.5 m s^−1^), and a water-retaining bulking matrix (75 °C, 7.4 mm, 0.5 m s^−1^). These findings demonstrate that convective drying thermodynamics can be strategically modulated to tailor avocado seed waste into specialized functional ingredients for the circular bioeconomy.

## 1. Introduction

Avocado ranks among the most economically important tropical fruit crops worldwide, with global production exceeding 9.3 million metric tons in 2024 [1]. Mexico is the largest producer, accounting for approximately 30% of global supply with around 2.8 million mt annually, of which more than 1.2 million mt are processed into derived products such as guacamole, pulp purees, and avocado oil [2]. This processing scale generates an estimated 300,000–400,000 mt of seed by-product per year in Mexico alone, representing a significant agro-industrial residue stream whose current disposal contributes to environmental pressure while its valorization potential remains largely untapped. Recovering these residues as functional food ingredients aligns with circular-bioeconomy approaches to convert agro-industrial waste into value-added products [3,4].

The avocado seed accounts for approximately 13–18% of the fresh fruit mass and contains a uniquely dense, lignocellulosic biomass rich in insoluble dietary fiber (>75% on a dry basis), resistant starch, and a diverse profile of bioactive compounds including chlorogenic acid, ferulic acid, catechins, and proanthocyanidins [5,6]. Aqueous and hydroalcoholic extracts of avocado seed exhibit antioxidant, anti-inflammatory, hypoglycemic, and antimicrobial activities at intake levels considered safe for human consumption [5,7]. This combination of high dietary fiber, bioactive compounds, and amphiphilic starch makes avocado seed a versatile raw material for multiple value-added applications: as a functional ingredient in bakery and beverage formulations [8]; as a source of food-grade pigments and natural dyes [9]; as a feedstock for bioplastic films [10]; and as a precursor for activated carbon and biofuel applications [11].

The high moisture content of the fresh seed nevertheless makes it highly perishable, so any of these applications requires a prior stabilization step. Convective hot-air drying is widely used in the food industry due to its simplicity, scalability, and compatibility with existing infrastructure [12]. This process couples simultaneous heat and mass transfer with irreversible physical and chemical changes in the material: prolonged thermal exposure can promote non-enzymatic browning, degrade phenolic compounds, and alter internal porosity, thereby modifying techno-functional properties such as water and oil retention [13,14].

Controlling temperature, air velocity, and sample thickness to accelerate moisture diffusion and preserve thermolabile bioactives may therefore come at the expense of the techno-functional profile [15], and vice versa, a trade-off that must be quantified before any of the applications listed above can be industrially deployed.

Although the chemical composition of avocado seed extracts [16] and the drying kinetics of fruit by-products [12,13,14] have been independently investigated, the simultaneous evaluation of mass-transfer thermodynamics, lignocellulosic fiber functionality, and thermolabile phytochemical stability within a single matrix has not been reported. In particular, no previous study has integrated empirical thin-layer kinetics, transport theory (*D*_eff_ and *h*_m_ via the heat-and-mass-transfer analogy), Principal Component Analysis, and multi-response desirability optimization to identify processing conditions tailored to distinct industrial end-uses of dehydrated avocado seed. The present study addresses this gap by providing a unified framework that links convective drying thermodynamics with the techno-functional and bioactive performance of the resulting powders.

## 2. Materials and Methods

### 2.1. Raw Material Preparation and Chemicals

Hass avocado (*Persea americana*) fruits at commercial physiological maturity were sourced from a processing facility located in Atapan, Michoacán, Mexico (19°26′ N, 102°03′ W) during the April 2024 harvest season. The seeds were manually separated from the mesocarp and pericarp, washed with distilled water to remove residual pulp, and superficially dried at ambient temperature. The fresh seeds were sliced into discs and ground using a commercial blender (OSTER BLSTVB-TV0-013, Atlanta, GA, USA) until a coarse, semimoist granulate mix was obtained. The initial moisture content was 52.4 ± 0.6% wet basis. The resulting material was vacuum-sealed in polyethylene bags and stored at 4 °C in the dark until thermal processing.

Analytical-grade solvents (methanol, ethanol, and acetone) and chromatographic standards (chlorogenic and ferulic acids) were supplied by CTR Scientific (Zapopan, Mexico). Reagents for the antioxidant assays, including 1,1-diphenyl-2-picrylhydrazyl (DPPH) and 2,2′-azino-bis(3-ethylbenzothiazoline-6-sulfonic acid) (ABTS), were acquired from Sigma-Aldrich (St. Louis, MO, USA).

### 2.2. Convective Drying Design

For each drying run, the ground seed material was placed into custom rectangular molds (25 × 20 cm internal dimensions) consisting of 3D-printed rigid frames that defined both the sample perimeter and the target sample thickness. Three sets of molds were used, with internal heights of 3, 6, and 9 mm, corresponding to the three levels of sample thickness. Each mold was positioned on a stainless-steel drying tray so that the tray formed the bottom surface of the cavity. The ground material was loaded into the mold cavity and leveled with a stainless-steel spatula so that the top surface matched the frame height, without external mechanical pressure. Sample thickness was therefore set exclusively by the internal height of the mold, while the corresponding wet mass required to fill each cavity was 100, 200, and 300 g for the 3, 6, and 9 mm treatments, respectively. The corresponding surface loads were 200, 400, and 600 g m^−2^, and the bulk density of the loaded material was approximately 0.67 g cm^−3^, uniform across all thicknesses. The loaded mold was placed as a single tray load in the drying chamber.

Dehydration experiments were performed in a convective tray dryer (Memmert UF160, Memmert GmbH + Co. KG, Schwabach, Germany) equipped with automated temperature and airflow controllers. A Box–Behnken Design (BBD) was implemented with three independent factors (Table 1): drying air temperature (45, 60, and 75 °C), sample thickness (3, 6, and 9 mm), and air velocity (0.5, 1.5, and 2.5 m s^−1^). The experimental matrix comprised 15 randomized runs, including three central points (60 °C, 6 mm, 1.5 m s^−1^) to evaluate reproducibility [14,17].

Drying was continuously monitored until the samples achieved a constant weight, corresponding to a final moisture content below 10% on a wet basis [18]. The dehydrated seed matrices were subsequently pulverized using a high-speed mill (IKA A10 basic, Staufen, Germany) and then passed through a 40-mesh screen (425 µm) to ensure particle-size uniformity. The resulting flours were packed in light-impermeable metallic bags and stored at −20 °C prior to analytical characterization.

### 2.3. Drying Kinetics and Mathematical Modeling

The experimental moisture ratio (*MR*) of the avocado seeds was calculated based on the dynamic, initial, and equilibrium moisture contents. To describe the mass transfer behavior, the experimental curves were fitted to six standard thin-layer kinetic models: Newton ($MR=\;e^{-kt}$), Page ($MR=\;e^{-kt^{n}}$), Modified Page ($MR=\;e^{-(k{t)}^{n}}$), Henderson and Pabis ($MR=\;ae^{-kt}$), Logarithmic ($MR=\;{ae}^{-kt}+c$), and Midilli ($MR=\;{ae}^{-kt^{n}}+bt$), where *a*, *b*, *c*, and *n* are empirical constants, *k* is the drying rate constant, and *t* is the drying time [19,20].

The kinetic parameters were estimated via non-linear least squares regression using the Levenberg–Marquardt algorithm. The predictive performance of the models was evaluated based on the coefficient of determination (R^2^), the mean squared error (MSE), the residual sum of squares (RSS), and the corrected Akaike Information Criterion (AICc) [21,22].

### 2.4. Moisture Transport Properties

The effective moisture diffusivity (*D*_eff_, m^2^ s^−1^) was quantified by fitting the experimental data to the analytical solution of Fick’s second law for an infinite slab geometry, assuming a uniform initial moisture distribution and negligible shrinkage during dehydration [16,23].

Furthermore, the external resistance to moisture removal at the boundary layer was evaluated through the convective mass transfer coefficient (*h*_m_, m s^−1^). To ensure independence from the empirical thin-layer kinetic models, *h*_m_ was determined using the heat and mass transfer analogy through dimensionless numbers [24]. The Sherwood number (*Sh*) for forced convection over a flat plate was calculated according to the following sequence of Equations (1)–(4):

(1) $$Re=\frac{\rho vL_{c}}{\mu }$$

(2) $$Sc=\frac{\mu }{\rho D_{v}}$$

(3) $$Sh=0.664\;{Re}^{0.5}\;{Sc}^{0.33}$$

(4) $$h_{m}=\frac{Sh\;D_{v}}{L_{c}}$$

where *Re* and *Sc* are the Reynolds and Schmidt numbers, respectively [25]. The properties of the drying air, density (ρ, kg m^−3^), dynamic viscosity (μ, kg m^−1^ s^−1^), and the diffusivity of water vapor in air (*D*_v_, m^2^ s^−1^), were evaluated at the specific drying temperatures. The term *v* represents the air velocity (m s^−1^), and *L*_c_ is the characteristic length of the sample (m).

### 2.5. Techno-Functional Capacity

Functional properties were determined following the standardized protocols for fruit fiber concentrates described by [26]. Swelling capacity (SWC) was assessed by hydrating 200 mg of the sample in 10 mL of distilled water for 18 h at 25 °C, recording the volume occupied by the swollen matrix per gram of dry sample (mL g^−1^ db).

For Water Retention Capacity (WRC), Solubility (SOL), and Oil Retention Capacity (ORC), samples (0.5 g) were dispersed in 10 mL of solvent (distilled water for WRC and SOL; commercial soybean oil for ORC). The suspensions were vortexed for 5 min, equilibrated for 18 h at room temperature, and centrifuged at 3125× *g* for 30 min (Gyrozen 1580 R, Gimpo, Republic of Korea). WRC (mL g^−1^ db) and SOL (%) were calculated from the weight of the wet pellet and the dried supernatant (60 °C for 24 h), respectively. ORC was calculated based on the unabsorbed free oil volume and expressed as mL of oil retained per gram of dry matter (mL g^−1^ db).

### 2.6. Colorimetric Assessment

Color measurements were performed with a Konica Minolta CR-410 chroma meter (CR-410, Konica Minolta Sensing, Tokyo, Japan) equipped with a pulsed xenon arc lamp as the illumination source and operating with a diffuse illumination/0° viewing geometry (d/0°) and a 50 mm measuring aperture. For the dehydrated seed powders, a CR-A50 granular materials attachment was used to ensure a representative reading over a controlled sample area. Measurements were referenced to the CIE Standard Illuminant D65 with the 2° Standard Observer. The instrument was calibrated against the manufacturer’s white calibration plate prior to each measurement session. For each sample, three independent readings were taken at different positions of the powder surface. To evaluate the visual quality, color saturation, and the extent of thermal damage, the Chroma (C*), Hue angle (*h*°), and Browning Index (BI) were mathematically derived from the fundamental L*, a*, and b* coordinates using the following Equations (5)–(7) [27,28]:

(5) $$C^{*}=\sqrt{a^{{*}^{2}}+b^{{*}^{2}}}$$

(6) $$h{}^{\circ}=arctan\left(\frac{b^{*}}{a^{*}}\right)$$

(7) $$BI=\frac{100\left(x-0.31\right)}{0.17}\;\;\mathrm{where}\;\;\;\;\;\;\;\;\;x=\frac{a^{*}+1.75L^{*}}{5.645L^{*}+a^{*}-3.012b^{*}}$$

### 2.7. Phenolic Content and Antioxidant Activity

Methanolic extracts were prepared by mixing 0.150 g of the seed flour with 25 mL of methanol. The mixture was vortexed and centrifuged at 4000× *g* for 15 min at 4 °C. The supernatant was filtered (0.45 µm) and recovered. The Total Phenolic Content (TPC) was quantified using the Folin–Ciocalteu method [29]. Absorbance was measured at 765 nm (Varioskan Lux, Thermo Scientific, Waltham, MA, USA), and results were expressed as mg of gallic acid equivalents per gram of dry matter (mg GAE g^−1^ db). A standard calibration curve was prepared with gallic acid at concentrations of 10–150 µg mL^−1^ in methanol. The resulting linear regression was Abs_765_ = 0.00423 × [gallic acid, mg L^−1^] − 0.0127, with a coefficient of determination of R^2^ = 0.998 (Supplementary Figure S2). All sample absorbances fell within the linear range of the curve.

Antioxidant activity was assessed using the DPPH and ABTS radical scavenging assays [30]. For the DPPH assay, 20 μL of the methanolic extract was mixed with 280 μL of a fresh methanolic DPPH solution and incubated in the dark at room temperature for 30 min. The absorbance was measured at 515 nm (Varioskan Lux, Thermo Scientific, Waltham, MA, USA). For the ABTS assay, the radical cation (ABTS^+^) was generated by reacting ABTS with potassium persulfate and allowing the mixture to stand in the dark for 16 h prior to use; the resulting solution was then diluted with methanol to an absorbance of 0.70 ± 0.02 at 734 nm. An aliquot of 10 μL of the methanolic extract was mixed with 290 μL of the ABTS^+^ working solution, incubated in the dark for 6 min, and the absorbance was measured at 734 nm (Varioskan Lux, Thermo Scientific, Waltham, MA, USA). In both assays, calibration curves were prepared with Trolox in methanol (DPPH: 1.25–50 mg L^−1^; ABTS: 0–62.5 mg L^−1^; R^2^ > 0.99), and results were expressed as milligrams of Trolox equivalents per gram of dry matter (mg TE g^−1^ db).

Individual phenolic compounds were profiled following the method described by [31]. Dried seed flour (500 mg) was extracted with 20 mL of methanol, vortexed, and centrifuged at 4000× *g* for 30 min at 4 °C. The supernatant was filtered (0.22 μm nylon) and injected (10 μL) into an Acquity Arc UHPLC-PDA system (Waters, Milford, MA, USA). Separation was performed on a Cortecs C18 reverse-phase column (4.6 mm × 150 mm, 2.7 μm pore size) at 40 °C, using a binary mobile phase system: solvent A was Milli-Q water acidified to pH 2.4 with orthophosphoric acid, and solvent B methanol. The flow rate was 1 mL min^−1^. A gradient elution was applied: 0–13.2 min (10–35% B), 13.2–15.6 min (35–98% B), and 15.6–19.2 min (98–10% B). Chlorogenic acid (CHA) and ferulic acid were identified and quantified at 320 nm using external calibration curves (2–200 ppm). Results were expressed as μg per gram of dry matter (μg g^−1^ db).

### 2.8. Multivariate Analysis and Multi-Response Optimization

To elucidate patterns and correlations between drying operational parameters and resulting quality attributes, Principal Component Analysis (PCA) was performed. Data were standardized (Z-scores) to account for differences in measurement scales, and the correlation matrix was used to extract the principal components, retaining only those with eigenvalues >1.

A multi-response optimization was subsequently conducted to identify the processing conditions that simultaneously maximize target phenolic compounds and optimize functional properties. The desirability function approach [17] was employed, mapping each individual response *i* into a dimensionless individual desirability *d*_i_ ranging from 0 (undesirable) to 1 (ideal) through Min–Max normalization. To limit the propagation of model uncertainty, particularly relevant for the responses with lower predicted R^2^, the normalization was applied to the observed experimental means at each design point rather than to model-predicted values. The global desirability *D* was then calculated as the geometric mean of the *n* individual desirabilities (Equation (8)):

(8) $$D={\left(\prod_{i=1}^{n}d_{i}\right)}^{\frac{1}{n}}$$

All individual desirabilities were weighted equally within each industrial scenario, as no prior knowledge justified a differential weighting. Three end-use scenarios were defined a priori (phytochemical concentrate, soluble ingredient, and hydration matrix), each with a distinct subset of responses to be maximized or minimized; the operational conditions maximizing *D* were reported as the optimum for each scenario.

### 2.9. Statistical Analysis

All physicochemical, colorimetric, phytochemical, and antioxidant analyses were performed in three analytical replicates per drying run, and the reported values represent the mean ± standard deviation of these analytical replicates.

Response-surface polynomial coefficients were estimated by ordinary least squares. The significance threshold for retaining individual terms was set at α = 0.05. Model adequacy was evaluated through lack-of-fit statistics and residual diagnostics (normality and homoscedasticity). For responses exhibiting significant lack-of-fit, reduced models were fitted by backward elimination retaining terms with *p* ≤ 0.10, and are reported in Supplementary Table S1. Internal cross-validation was performed at the central point of the design; full diagnostic outputs are provided in Supplementary Tables S1–S3 and Figures S1 and S3. All statistical analyses, including non-linear regression of the thin-layer kinetic models, correlation analysis, principal component analysis, and desirability optimization, were performed in Python (version 3.12.13, Python Software Foundation, Wilmington, DE, USA) using NumPy, pandas, SciPy (curve_fit and pearsonr functions), and scikit-learn (StandardScaler and PCA modules), executed in Google Colaboratory (Google LLC, Mountain View, CA, USA). Figures were generated using Matplotlib and Seaborn in the same environment.

## 3. Results and Discussion

Before discussing the individual responses, we note that several of the fitted response surface models exhibited a statistically significant lack-of-fit (LoF). For responses where significant LoF persisted even after applying backward elimination (Supplementary Table S1), the variability among analytical replicates of the same treatment was found to be comparable in magnitude to the variability explained by the polynomial across the experimental domain. This inflates the LoF F-statistic and is attributable to the intrinsic biological heterogeneity of the avocado seed tissue, which exceeds the resolution of a second-order polynomial within the explored range. The directional information provided by the models (that is, the sign and ranking of the operational effects) remains internally consistent, as evidenced by the internal cross-validation results (Supplementary Table S3), and is used here for process-optimization purposes rather than as an absolute predictive tool.

### 3.1. Drying Curves and Kinetic Mode Fitting

**Figure 1:**
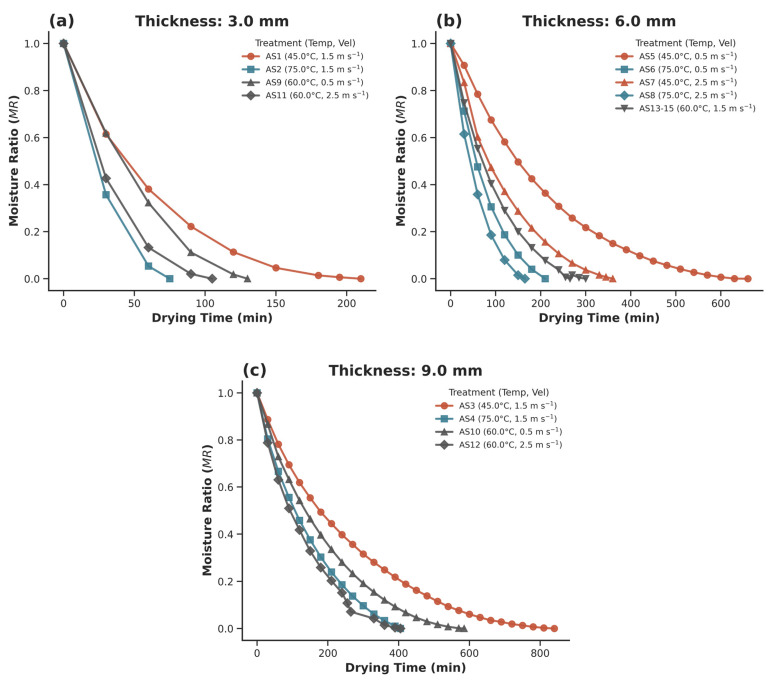
Experimental drying kinetics of avocado seeds (*Persea americana*) under convective drying. The curves represent the moisture ratio (MR) evolution over time for samples with varying thicknesses: (**a**) 3 mm, (**b**) 6 mm, and (**c**) 9 mm. Treatments were evaluated at different temperatures (45, 60, and 75 °C) and air velocities (0.5, 1.5, and 2.5 m s^−1^). Central point treatments (60 °C, 6 mm, 1.5 m s^−1^) are presented as a single averaged curve to illustrate the baseline process reproducibility.

The experimental drying curves of avocado seeds, expressed as the evolution of the moisture ratio (*MR*) over time under varying convective conditions, are presented in Figure 1. The dehydration profiles exhibited a continuous decay characterized by a falling-rate period across all evaluated treatments. The absence of a constant drying rate period indicates that internal moisture diffusion toward the surface was the primary mass transfer mechanism governing the dehydration of the matrix, a physical phenomenon common in dense plant tissues subjected to convective drying [16,19].

The operational parameters showed a significant effect on the time required to reach a stabilized moisture content (<10% wb). Sample thickness acted as the main physical barrier to mass transfer. Thin slabs (3 mm) exhibited the fastest dehydration rates, requiring between 75 and 210 min depending on the thermal and airflow conditions (Figure 1a). Increasing the geometry to 9 mm substantially prolonged the process, requiring up to 840 min at 45 °C to fully dry (Figure 1c), as the internal water had to migrate through a more extensive structural network. Furthermore, increasing the drying air temperature from 45 to 75 °C accelerated the kinetic profiles. Elevated thermal gradients increase the kinetic energy of water molecules, raising the vapor pressure within the seed tissue and promoting a faster evaporation rate toward the convective boundary layer [12,32].

**Figure 2:**
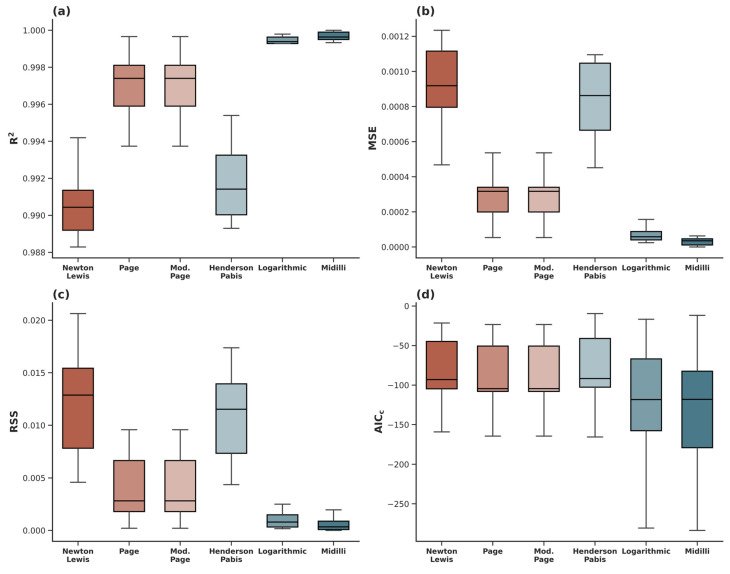
Statistical goodness-of-fit evaluation for the thin-layer mathematical models. The boxplots illustrate the overall performance and error distribution of the six evaluated kinetic models across all experimental conditions. The panels display: (**a**) the coefficient of determination ($R^{2}$), (**b**) the mean squared error (MSE), (**c**) the residual sum of squares (RSS), and (**d**) the corrected Akaike information criterion (AICc). Lower dispersion and values closer to the ideal targets indicate superior model reliability.

To quantify this behavior, the experimental *MR* data were fitted to six thin-layer models. The statistical performance of each equation is summarized in Figure 2. Although all evaluated models captured the general dehydration trend (R^2^ > 0.90), the Midilli model demonstrated the highest predictive accuracy across the experimental domain. The Midilli equation exhibited the most compact interquartile range, maximized R^2^ values (0.998–1.0), minimized error functions (MSE and RSS), and yielded the most negative AICc values, which indicates an accurate prediction while preventing mathematical overfitting [20].

The suitability of the Midilli model is linked to the anatomical composition of the avocado seed, which is predominantly composed of intracellular starch and a dense network of insoluble dietary fiber [3]. These structural components form a compact matrix that traps bound water, causing a pronounced resistance to capillary diffusion during the final stages of the process. The empirical parameters of the Midilli model provided the mathematical flexibility required to predict this prolonged dehydration behavior accurately [16,33].

**Figure 3:**
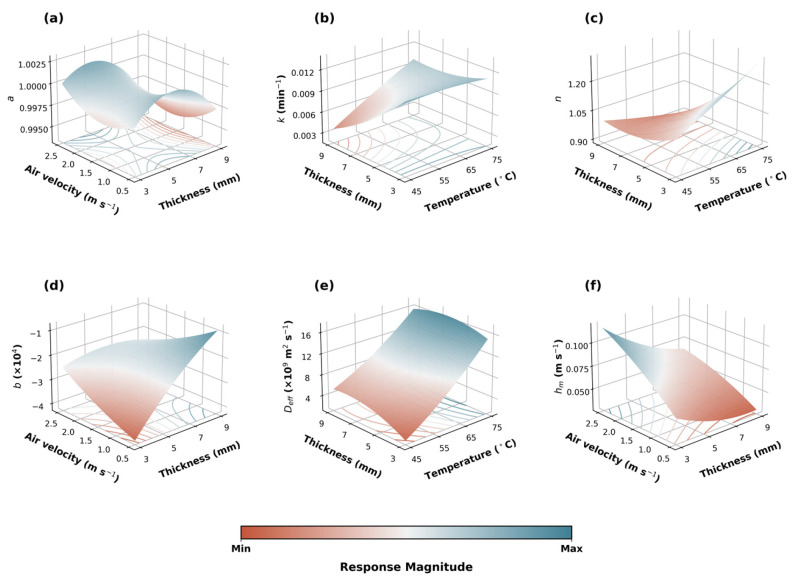
Response surface methodology (RSM) analysis of the kinetic parameters and mass transfer phenomena. The 3D plots illustrate the interactive effects of temperature and sample thickness (at constant air velocity of 1.5 m s^−1^) on the empirical constants of the Midilli model: parameter *a* (**a**), drying rate constant *k* (**b**), and the dimensionless parameters *n* (**c**), and *b* (**d**). Mass transfer parameters are depicted through the effective moisture diffusivity, *D*_eff_ (**e**), and the convective mass transfer coefficient, *h*_m_ (**f**).

An analysis of variance (ANOVA) performed on the empirical constants of the Midilli model (Table 2) showed that the drying rate constant (*k*) was significantly (*p* < 0.05) affected by air temperature, sample thickness, and air velocity. A significant interactive effect between temperature and thickness (*p* < 0.001) on the drying constant *k* was also observed. As visualized in Figure 3, the thermal acceleration of the drying rate was more pronounced in thinner samples. In contrast, thick samples (9 mm) increased resistance to heat and mass transfer, regardless of the elevated temperatures applied [21].

### 3.2. Effective Moisture Diffusivity and Mass Transfer Coefficients

The dehydration efficiency of solid matrices relies on two consecutive transport phenomena: the internal migration of water towards the surface, quantified by the effective moisture diffusivity (*D*_eff_), and the evaporation into the drying air stream, defined by the convective mass transfer coefficient (*h*_m_) [12].

The ANOVA of the second-order polynomial model for *D*_eff_ (Table 2) showed high predictive accuracy (R^2^ = 0.954). Internal moisture diffusion was highly dependent on air temperature, sample thickness, and air velocity (*p* < 0.001). The calculated *D*_eff_ values for the avocado seeds ranged within the order of ×10^−9^ m^2^ s^−1^, which is consistent with the characteristic diffusivity intervals reported for dense, starch-rich agricultural products [16,23].

As observed in the response surface plot (Figure 3e), increasing the drying temperature enhanced the effective diffusivity. From a thermodynamic perspective, elevated thermal profiles increase the enthalpy of the system, reducing the viscosity of the internal liquid water while breaking hydrogen bonds between water molecules and the amylose and amylopectin chains in the seed starch [5]. This facilitates moisture migration towards the surface [19]. Additionally, the slight increase in apparent *D*_eff_ for thicker geometries (9 mm) can be attributed to the larger internal moisture gradients developed over extended characteristic lengths [21].

The convective mass transfer coefficient (*h*_m_) characterizes the external resistance to moisture removal. The quadratic regression model for *h*_m_ exhibited an accurate fit to the experimental data (R^2^ = 0.998). Air velocity and sample thickness were the dominant linear factors governing this parameter (*p* < 0.001), alongside their antagonistic interaction (*p* < 0.001). As depicted in Figure 3f, *h*_m_ reached its maximum values at the highest air velocity (2.5 m s^−1^) combined with the thinnest geometry (3 mm). High air velocities continuously sweep the evaporated moisture from the seed surface, reducing boundary layer resistance and enhancing moisture removal [24]. Conversely, bulkier structures distort the aerodynamic flow of the drying air across the tray, dampening convective moisture removal [34].

### 3.3. Techno-Functional Properties

The techno-functional properties of dehydrated dietary fiber concentrates dictate their physicochemical behavior and potential application as food ingredients. The ANOVA (Table 3) revealed that convective drying parameters exerted varying degrees of influence over the hydration and oil-binding capacities of the resulting flours.

The Swelling Capacity (SWC) and Oil Retention Capacity (ORC) exhibited lower coefficients of determination (R^2^ = 0.269 and 0.381, respectively in Table 3; R^2^adj = 0.20 and 0.25 after backward elimination of non-significant terms in Supplementary Table S1). This limited predictive power indicates that the operational variables explain only a small fraction of the observed variability for these two responses. For SWC, only temperature retained a significant linear effect (*p* = 0.003), while ORC responded significantly to the quadratic effect of temperature (*p* = 0.001), consistent with a partial exposure of lipophilic binding sites at intermediate thermal loads that collapses at temperature extremes [35,36]. Rather than reflecting an intrinsic instability of the fiber matrix, this low sensitivity to the operational variables is more parsimoniously interpreted as evidence that the lignocellulosic skeleton of the seed, dominated by cellulose and lignin, is largely resilient to the drying conditions tested. This interpretation is supported by direct quantitative evidence from the retention analysis against the freeze-dried reference (Supplementary Table S4), which shows that SWC was preserved within 93–113% and ORC within 67–86% across all 15 treatments. The fiber matrix therefore appears advantageous for formulating lipid-binding functional foods, as its baseline porosity and hydration architecture are preserved even under intense convective conditions [3,10,37].

**Figure 4:**
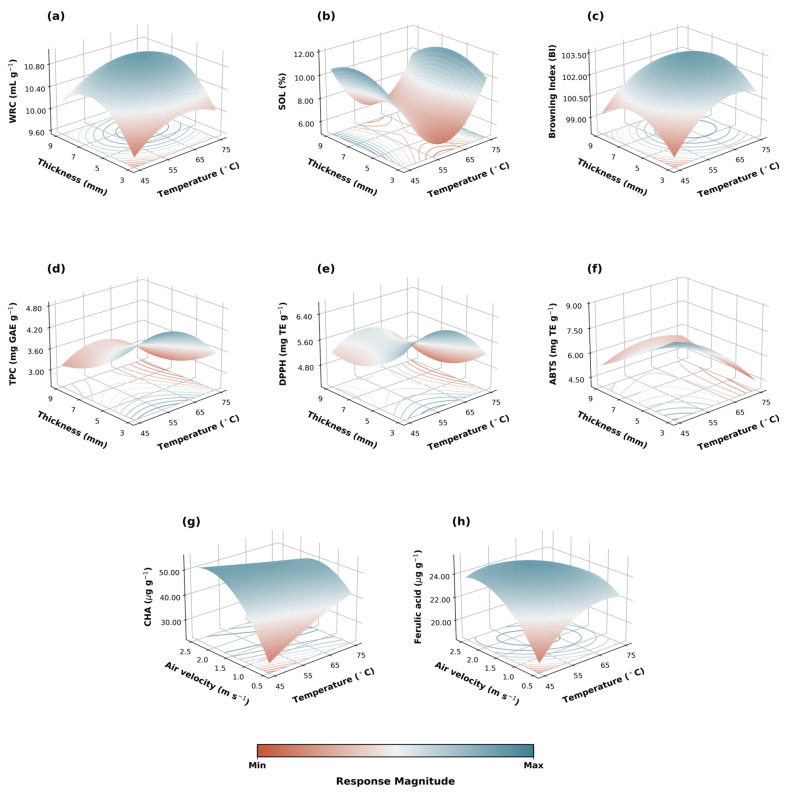
Response surface plots for functional and phytochemical properties of avocado seed powder. The 3D surfaces visualize the interactive effects of temperature and sample thickness on: (**a**) Water retention capacity (WRC), (**b**) Solubility (SOL), (**c**) Browning index (BI), (**d**) Total phenolic content (TPC), (**e**) DPPH radical scavenging activity, (**f**) ABTS antioxidant capacity, (**g**) Chlorogenic acid content (CHA), and (**h**) Ferulic acid content. Note: SWC, ORC, and alternative colorimetric vectors (C*, *h*°, Δ*E*) were excluded due to their lower statistical sensitivity to the thermal gradients.

Conversely, the Water Retention Capacity (WRC) and Solubility (SOL) were sensitive to processing conditions. The model for WRC (R^2^ = 0.577) indicated significant linear effects for sample thickness (*p* = 0.031) and air velocity (*p* = 0.023). As observed in the response surface for WRC (Figure 4a), variations in geometry and air velocity dictate the drying exposure time, which induces macroscopic shrinkage and alters the internal pore volume available to trap water molecules physically [13,38]. Notably, WRC retention against the freeze-dried reference reached 148–180% across all treatments (Supplementary Table S4), indicating that convective drying does not merely preserve but actively enhances water binding, a finding with direct implications for hydration-focused ingredient design.

Solubility emerged as the most responsive functional parameter (R^2^ = 0.835, *p* < 0.001). As visualized in Figure 4b, SOL exhibited a parabolic trend driven by the quadratic effects of temperature, thickness, and air velocity (*p* < 0.001). At intermediate temperatures, the thermal energy may promote partial gelatinization of starch granules and depolymerization of the insoluble protopectin fraction, enhancing the extractability of short-chain carbohydrates [39,40]. However, under extreme thermal loads and prolonged drying times, solubility decreased. These observations are consistent with case-hardening phenomena, although no direct structural characterization was performed, where rapid surface dehydration collapses capillary pores and creates an impermeable crust, while prolonged heat triggers Maillard cross-linking that traps soluble fractions within the matrix [41].

### 3.4. Colorimetric Changes

Surface color serves as a physical indicator of thermal degradation during food processing [42]. Color changes in the dehydrated seed flours were first interpreted in the classic CIELab framework relative to the freeze-dried reference (L* = 45.92, a* = 12.00, b* = 24.98). All convectively dried treatments showed a moderate de-crease in lightness, with L* values ranging from 42.98 (AS8) to 45.18 (AS7), consistent with a darkening of the powder driven by the development of brown pigments. The a* co-ordinate (green–red axis) consistently decreased by 6–14% relative to the reference (a* = 10.23–11.19 across treatments), indicating a subtle shift toward less reddish tones, while the b* coordinate (blue–yellow axis) was largely preserved or slightly increased (b* = 24.50–26.85), reflecting retention of the yellow-amber character of the powder. The vector combination of these changes, moderate darkening, slight loss of redness, and stable yellowness, is visually perceivable as a transition from a bright amber-orange (freeze-dried) toward a deeper amber-brown (convectively dried), particularly at the highest thermal loads.

To move beyond descriptive interpretation, the CIELab-derived parameters (Chroma C*, Hue angle *h*°, Browning Index BI, and total color difference Δ*E*) were fitted to the Box–Behnken design (Table 4). Model performance differed markedly among the four indicators. Chroma (C*) exhibited a moderate coefficient of determination (R^2^ = 0.488) driven principally by the linear effect of air velocity (*p* < 0.001), suggesting that rapid convective moisture removal helps preserve natural color saturation before extended heat exposure degrades native pigments. The Browning Index (BI), which quantifies the development of brown color associated with thermal damage, yielded a significant regression model (R^2^ = 0.696, *p* < 0.001), driven linearly by temperature (*p* = 0.002) and air velocity (*p* < 0.001). As observed in Figure 4c, browning values increased at 75 °C and prolonged drying times (thicker samples). This darkening is consistent with non-enzymatic browning via the Maillard reaction; specific reaction products were not quantified [43]. High air velocities (2.5 m s^−1^) mitigated the browning effect by rapidly pushing the sample past the intermediate moisture zone, reducing the time available for Maillard reactions to occur [44].

In contrast, Hue angle (*h*°, R^2^ = 0.352) and the total color difference (Δ*E*, R^2^ = 0.171) were not modeled robustly, indicating that these vectors fluctuated mainly due to the inherent biological variability of the seed tissue rather than to systematic effects of the operational variables. After backward elimination, the polynomial model for Δ*E* retained no statistically significant terms (Supplementary Table S1), so Δ*E* was excluded from the multi-response optimization (Section 3.7); its values are nevertheless reported descriptively in Table 4 to characterize the overall color change in each treatment relative to the lyophilized reference.

### 3.5. Phytochemical Profiling and Antioxidant Activity

The retention of phenolic compounds is a critical target when valorizing agro-industrial by-products. The ANOVA results (Table 5) demonstrated that convective drying parameters modulated the phytochemical integrity of the seed matrix. The Total Phenolic Content (TPC) and the antioxidant capacities (DPPH and ABTS) were mathematically modeled with high statistical precision (R^2^ ranging from 0.829 to 0.939). For these general indicators, all three operational factors exerted significant linear effects (*p* < 0.001).

As illustrated in the response surfaces (Figure 4d–f), TPC degradation and antioxidant activity occurred as both temperature and sample thickness increased. Thicker geometries (9 mm) extended the drying time up to 840 min, exposing the internal phenolic compounds to a prolonged cumulative thermal load. This aligns with the behavior reported by [14], confirming that minimizing total thermal exposure time is necessary for phenolic preservation. High air velocities exhibited a protective effect (*p* < 0.001), as rapid moisture evaporation induces localized evaporative cooling, maintaining the internal tissue temperature lower than the drying air during the early stages of dehydration [28].

This time–temperature dependence was further analyzed by UHPLC profiling of specific phenolic markers. The models for chlorogenic acid (CHA) and ferulic acid revealed that the linear effect of temperature alone was not significant (*p* > 0.50). Instead, the degradation of these acids was penalized by sample thickness (*p* = 0.005 and *p* = 0.002) and protected by air velocity (*p* = 0.002 and *p* = 0.003). A significant interaction between temperature and air velocity (*p* < 0.01) was observed for both compounds (Figure 4g,h). A fraction of phenolic compounds in dense seeds is bound to cell wall fibers via ester bonds [45]. The initial thermal exposure may promote hydrolysis of linkages, releasing the bound phenolic compounds into their free forms [46]. However, their subsequent survival depends on the overall processing time. Prolonged thermal exposure, inherent to thicker samples with slow internal moisture diffusion, could be associated with oxidative degradation, a hypothesis that would require fractionation of free and bound phenolic forms to be confirmed [15].

To enable a quantitative interpretation of the bioactive retention, the phytochemical content of all 15 treatments was expressed as a percentage of the corresponding value in the freeze-dried reference AS0 (Supplementary Table S4; AS0 reference values: TPC = 4.288 mg GAE g^−1^, chlorogenic acid = 50.02 µg g^−1^, ferulic acid = 21.53 µg g^−1^). The total phenolic content (TPC) retention ranged from 67% to 120% (mean 86%), with four treatments exceeding 100%—most notably AS11 (120%), AS9 (117%), AS1 (104%), and AS7 (102%). The chlorogenic acid (CHA) retention reached up to 127% at AS7 (45 °C, 6 mm, 2.5 m s^−1^) and 119% at AS2 (75 °C, 3 mm, 1.5 m s^−1^). Most strikingly, ferulic acid retention exceeded 100% in 10 of the 15 treatments, with a mean of 105% and a maximum of 117%. These observations provide direct quantitative evidence supporting the previously hypothesized thermal liberation of phenolic acids that are esterified to the lignocellulosic cell-wall matrix: in samples where the thermal exposure was sufficient to disrupt the ester linkages but not prolonged enough to degrade the released compounds, the extractable phenolic content can exceed that of the unprocessed lyophilized reference. The particularly strong effect on ferulic acid is consistent with its well-documented covalent linkage to arabinoxylans and lignin in plant cell walls [45], which is more efficiently disrupted by convective heating than by ice sublimation during lyophilization.

In contrast, the antioxidant capacities measured by the DPPH (mean retention 81%) and ABTS (mean retention 47%) assays were consistently below 100%, indicating that a fraction of the freeze-dried antioxidant activity was lost during convective drying. The substantially lower ABTS retention suggests that the ABTS-active hydrophilic phenolics are more thermolabile than the DPPH-active fraction. This loss must be considered together with the gain in extractable phenolic compounds: the net effect on the bioactive profile depends on the intended application of the dried seed powder.

### 3.6. Multivariate Analysis: 3D-PCA Integration

**Figure 5:**
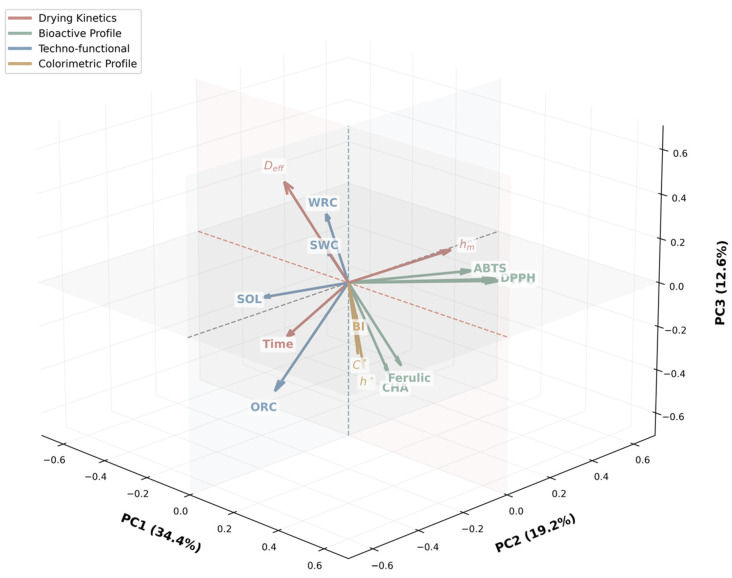
Three-dimensional Principal Component Analysis (3D-PCA) biplot. The projection illustrates the spatial distribution and inter-variable correlations of the 15 physicochemical and kinetic parameters across PC1, PC2, and PC3. Vectors represent the direction and magnitude of the contribution of each variable to the total explained variance, highlighting the clustering of phenolic compounds versus mass transfer kinetics.

To assess the impact of convective drying conditions on the evaluated properties of the avocado seed matrix, a Principal Component Analysis (PCA) was performed on the standardized values of 15 variables (Figure 5) grouped into four conceptual blocks: (i) drying kinetics: total drying time, effective moisture diffusivity (*D*_eff_), and convective mass transfer coefficient (*h*_m_); (ii) colorimetric profile: Chroma (C), Hue angle (*h*°), and Browning Index (BI); (iii) bioactive profile: Total Phenolic Content (TPC), DPPH and ABTS radical scavenging capacities, chlorogenic acid (CHA), and ferulic acid; and (iv) techno-functional profile: Swelling Capacity (SWC), Oil Retention Capacity (ORC), Water Retention Capacity (WRC), and Solubility (SOL). The model explained 66.12% of the total experimental variance across its first three principal components.

The first principal component (PC1, 34.37%) was driven by mass transfer kinetics, showing an inverse relationship between the convective mass transfer coefficient (*h*_m_) and the total drying time. Fast-drying treatments are projected along the positive axis of PC1, associated with the preservation of total phenolics and the rapid release of bound phenolic acids [45,46].

The second principal component (PC2, 19.19%) elucidated the kinetic-quality relationship. The bioactive cluster (TPC, DPPH, ABTS) was positioned antagonistically to the Browning Index (BI). This separation confirms that while mass transfer (PC1) dictates overall speed, the preservation of polyphenols is opposed to the accumulation of Maillard-derived brown pigments (PC2), which require prolonged thermal loads [14,43]. A positive correlation between Hue angle (*h*°) and chlorogenic acid retention (r = 0.730, *p* < 0.001) indicated that superficial color parameters could serve as a non-destructive proxy to monitor the integrity of phenolic acids during thermal processing [42,44].

The techno-functional properties (WRC, SWC, ORC) dominated the third principal component (PC3, 12.56%). This specific spatial orientation, orthogonal to the bioactive degradation pathways, reflects the relative thermal resilience of the lignocellulosic matrix [26,36]. The hydration capacity of the avocado seed fibers responded independently to the processing conditions compared to the phytochemical profile.

### 3.7. Multi-Scenario Technical Optimization and Industrial Potential

A multi-response Desirability Function (DF) was implemented to establish optimal processing conditions tailored for specific applications (Table 6). The robustness of the predictive models, evidenced by a difference of less than 0.20 between the adjusted and predicted coefficients of determination (R^2^adj vs. R^2^pred), provided a statistical foundation for these projections.

**Figure 6:**
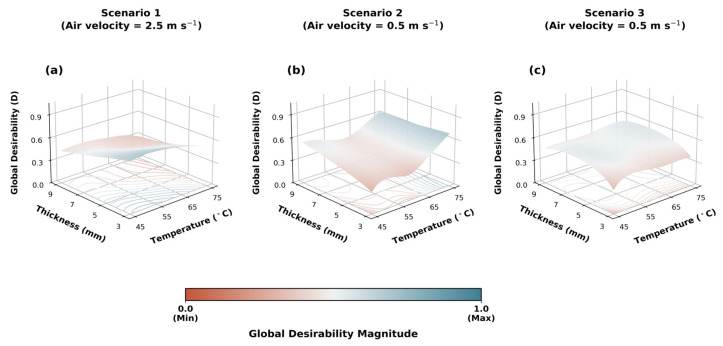
Global desirability (*D*) response surfaces for multi-scenario process optimization. The 3D surfaces illustrate the interaction between temperature and thickness under scenario-specific air velocities: (**a**) Scenario 1: Phytochemical extract optimization (fixed at 2.5 m s^−1^), (**b**) Scenario 2: Solubility-swelling profile (fixed at 0.5 m s^−1^), and (**c**) Scenario 3: Hydration-retention matrix (fixed at 0.5 m s^−1^).

Scenario 1 (“Phytochemical Extract”) was formulated to maximize the retention of phytochemicals. The global desirability reached its maximum (*D* = 0.79) at 45 °C, 3 mm thickness, and 2.5 m s^−1^ air velocity (Figure 6a). This operational window minimizes the cumulative thermal load, predicting a maximum CHA retention of 55.82 µg g^−1^. Rapid aerodynamic moisture removal at low thermal gradients favors bioactive preservation [15].

Scenario 2 (“Solubility Profile”) focused on optimizing hydration metrics in liquid food systems. The optimum shifted to 75 °C, 8.7 mm, and 0.5 m s^−1^ (*D* = 0.72, Figure 6b). Prolonged heat exposure is consistent with a thermally induced softening of the matrix, potentially involving partial gelatinization of intracellular starch and depolymerization of insoluble pectins, increasing the surface area for swelling and water solubility [5,37].

Scenario 3 (“Hydration Matrix”) prioritized WRC and ORC for moisture-sensitive bulking matrices. The optimum stabilized at 75 °C, 7.4 mm, and 0.5 m s^−1^ (*D* = 0.62, Figure 6c), predicting a WRC of 10.55 mL g^−1^. The dense seed matrix requires sustained thermal intensity to expand internal structural capillaries without reaching the threshold of severe case-hardening [13,38].

**Figure 7:**
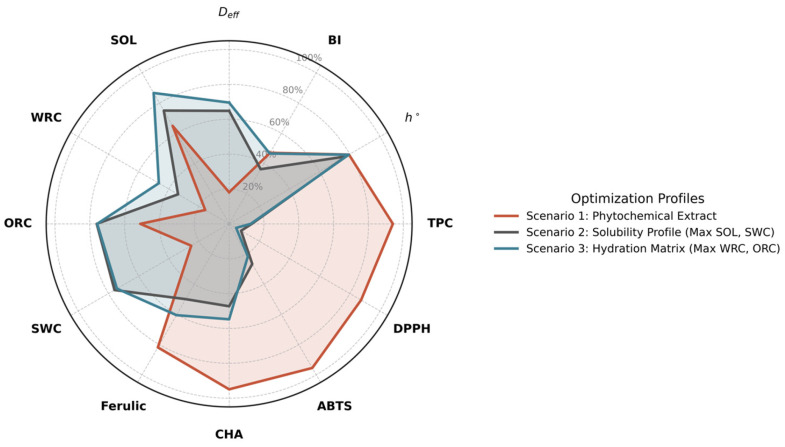
Optimization fingerprints (Radar Chart) for multi-response technical profiles. The chart compares the normalized predicted values for the three technical scenarios: Phytochemical Extract (Red), Solubility Profile (Grey), and Hydration Matrix (Blue). Each axis represents one of the 12 performance variables, enabling direct comparison of the trade-offs between drying efficiency and functional property retention.

The multi-response fingerprint generated in the radar plot (Figure 7) visually confirms that avocado seeds can be processed into different technical grades. Convective drying conditions can be modulated to produce specialized functional ingredients based on their final industrial application [4].

#### Industrial Considerations and Outlook

Translating these results to industrial implementation requires consideration of several practical and regulatory factors. From an energy standpoint, a first-order estimation of the specific energy consumption at laboratory scale (based on the nominal rated power of the dryer, the loaded wet mass, and the model-predicted drying times) indicates that Scenarios 1, 2, and 3 would require on the order of 75, 69, and 61 kWh kg^−1^ dry product, respectively. A counter-intuitive insight emerges: the low-temperature scenario consumes approximately 24% more energy per kg of dry product than the high-temperature scenario, because the longer drying time offsets the lower thermal load. Industrial-scale dryers with heat-recovery systems would reduce these absolute values substantially, but the qualitative trade-off would be preserved.

Beyond energy, convective tray drying is a well-established unit operation in the food industry, with commercial-scale configurations already available (continuous belt, multi-pass tray, fluidized bed), so the processing strategy proposed here is compatible with existing infrastructure in avocado-processing facilities without requiring novel equipment. Regarding product safety, all 15 treatments reached a final moisture content below 10% w.b., which for lignocellulosic seed matrices of this composition typically corresponds to a water activity well below the threshold of a_w_ ≤ 0.6 commonly associated with the microbiological stability of dehydrated plant ingredients [47]. This supports the shelf-stability of the dried seed powders against both vegetative pathogens and fungal spoilage. Avocado seeds are known to contain trace levels of cyanogenic glycosides, hydrolysable tannins, and persenone A; however, published toxicological studies indicate that the levels present in mature Hass seeds are well below toxicity thresholds for human consumption at typical food-ingredient inclusion levels (≤5–10% of formulation) [5,7], and the thermal exposure during convective drying is expected to further reduce the thermolabile fraction, particularly cyanogenic glycosides, although their direct quantification was beyond the scope of the present work and should be addressed prior to commercial implementation.

From a regulatory perspective, avocado seed is not currently included in the U.S. FDA GRAS list, nor authorized as a novel food in the European Union under Regulation (EU) 2015/2283 [48]. Commercial use as a food ingredient would therefore require either the demonstration of a history of safe consumption in a specific population or a novel-food dossier substantiating safety, while in Mexico, derivatives of food matrices fall under NOM-251-SSA1-2009 [49] and require Good Manufacturing Practice compliance. These considerations define the regulatory pathway for commercialization rather than acting as absolute barriers, and the present study provides the technical foundation for that path.

Although the optimized conditions were derived from statistically validated models and supported by internal cross-validation at the central point, experimental confirmation of the three predicted scenarios was beyond the scope of this study and is identified as a priority for future work, particularly to validate the techno-functional performance of Scenarios 2 and 3 in real food matrices. Biological replication across independent avocado harvests is similarly recognized as a limitation of the present design.

## 4. Conclusions

This study characterized the convective drying of Hass avocado seed by-products across a Box–Behnken design spanning temperature (45–75 °C), sample thickness (3–9 mm), and air velocity (0.5–2.5 m s^−1^), coupling empirical thin-layer kinetics with transport theory, principal component analysis, and multi-response desirability optimization. Drying was accurately described by the Midilli model (R^2^ > 0.998), with effective moisture diffusivities of 4.6–15.7 × 10^−9^ m^2^ s^−1^ and convective mass transfer coefficients of 0.04–0.10 m s^−1^. Sample thickness emerged as the dominant kinetic factor (standardized |t| = 37.8), followed by temperature, a dependence that propagated to most quality-related responses examined.

The bioactive profile was influenced by drying conditions in a compound-specific manner. Relative to the freeze-dried reference, total phenolic content was retained within 67–120% across treatments, with four treatments exceeding the lyophilized control due to the thermal liberation of cell-wall-bound phenolics. Chlorogenic acid retention reached 127% under short, thin-layer conditions, and ferulic acid retention exceeded 100% in 10 of 15 treatments (mean 105%), providing direct quantitative evidence of the release of phenolic acids from the lignocellulosic matrix. Techno-functional properties were largely preserved or enhanced: water retention capacity systematically exceeded the lyophilized control by 48–80%, positioning convective drying as a viable, and in some cases preferable, alternative to lyophilization for hydration-focused applications; swelling capacity was preserved within 93–113%, and oil retention capacity was moderately reduced (67–86%).

The multi-response desirability optimization identified three industrially distinct end-use scenarios: a phytochemical-concentrate profile (45 °C, 3 mm, 2.5 m s^−1^; D = 0.79), a soluble-ingredient profile (75 °C, 8.7 mm, 0.5 m s^−1^; D = 0.72), and a water-retaining matrix profile (75 °C, 7.4 mm, 0.5 m s^−1^; D = 0.62). The specific energy consumption estimated at laboratory scale ranged on the order of 60.7 to 75.3 kWh kg^−1^ dry product, with the counter-intuitive finding that low-temperature processing requires approximately 24% more energy per kg of dry product than the high-temperature route because the longer drying time offsets the lower thermal load, an insight of direct relevance to industrial cost analyses.

Overall, the results support the use of convective drying as a scalable stabilization strategy for avocado seed by-products, with processing conditions tuned to the intended end use rather than to a single global optimum. Priority directions for future work include the experimental validation of the three optimized scenarios at pilot scale, direct microstructural characterization to test the inferential mechanistic interpretations presented here (case-hardening, starch gelatinization, cell-wall disruption), quantification of antinutritional compounds and their evolution during drying to support the regulatory pathway toward food-grade applications, and bioaccessibility studies of the retained phenolic acids in simulated gastrointestinal models to confirm their functional relevance for human nutrition.

## Figures and Tables

**Table 1:** Box–Behnken design for the convective drying of avocado seed.

Run	Treatment	X_1_	X_2_	X_3_	Temperature (°C)	Thickness (mm)	Air Velocity (m s^−1^)
1	AS1	−1	−1	0	45	3	1.5
2	AS2	1	−1	0	75	3	1.5
3	AS3	−1	1	0	45	9	1.5
4	AS4	1	1	0	75	9	1.5
5	AS5	−1	0	−1	45	6	0.5
6	AS6	1	0	−1	75	6	0.5
7	AS7	−1	0	1	45	6	2.5
8	AS8	1	0	1	75	6	2.5
9	AS9	0	−1	−1	60	3	0.5
10	AS10	0	1	−1	60	9	0.5
11	AS11	0	−1	1	60	3	2.5
12	AS12	0	1	1	60	9	2.5
13	AS13	0	0	0	60	6	1.5
14	AS14	0	0	0	60	6	1.5
15	AS15	0	0	0	60	6	1.5

**Table 2:** Analysis of variance (ANOVA) of the second-order polynomial models for the drying kinetics and mass transfer parameters.

Model Parameters	Midilli Parameters									Moisture Transport Parameters		
	*a*	(*p*-Value)	*k*		*n*		*b* (×10^−4^)		*D*_eff_ (×10^−9^)	(*p*-Value)	*h*_m_	
Linear coefficient	0.999	<0.001 ***	0.009	<0.001 ***	1.025	<0.001 ***	−2.343	<0.001 ***	10.0313	<0.001 ***	0.064	<0.001 ***
X_1_ (Temperature—°C)	−0.002	<0.001 ***	0.001	<0.001 ***	0.036	<0.001 ***	−1.159	<0.001 ***	6.0238	<0.001 ***	0.003	<0.001 ***
X_2_ (Thickness—mm)	−0.001	<0.001 ***	−0.002	<0.001 ***	−0.119	<0.001 ***	0.837	<0.001 ***	0.9799	<0.001 ***	−0.019	<0.001 ***
X_3_ (Air velocity—m s^−1^)	0.000	0.994	0.002	<0.001 ***	−0.022	<0.001 ***	0.033	0.678	2.1370	<0.001 ***	0.024	<0.001 ***
X_1_^2^	0.002	<0.001 ***	0.000	0.674	0.006	0.518	0.138	0.238	0.8887	0.020 *	0.000	0.501
X_2_^2^	−0.003	<0.001 ***	0.001	0.064	0.038	<0.001 ***	−0.258	0.031 *	−1.3169	<0.001 ***	0.007	<0.001 ***
X_3_^2^	0.002	<0.001 ***	−0.001	0.001 **	0.056	<0.001 ***	0.131	0.262	1.6818	<0.001 ***	−0.004	<0.001 ***
X_1_X_2_	0.000	0.515	0.002	<0.001 ***	−0.084	<0.001 ***	0.152	0.178	−0.7170	0.049 *	−0.001	0.012 *
X_1_X_3_	−0.001	0.208	0.000	0.693	0.007	0.386	0.039	0.728	0.2854	0.422	0.001	0.003 **
X_2_X_3_	−0.001	0.154	−0.001	0.008 **	0.003	0.755	−0.791	<0.001 ***	0.7894	0.031 *	−0.007	<0.001 ***
Model		<0.001 ***		<0.001 ***		<0.001 ***		<0.001 ***		<0.001 ***		<0.001 ***
Linear		<0.001 ***		<0.001 ***		<0.001 ***		<0.001 ***		<0.001 ***		<0.001 ***
Quadratic		<0.001 ***		0.003 **		<0.001 ***		0.054		<0.001 ***		<0.001 ***
Interaction		0.259		<0.001 ***		<0.001 ***		<0.001 ***		0.032 *		<0.001 ***
*R* ^2^	77.13%		86.89%		94.55%		91.88%		95.43%		99.78%	
Adj *R*^2^	71.25%		83.52%		93.15%		89.79%		94.25%		99.72%	
Predicted *R*^2^	60.06%		78.25%		90.92%		85.98%		92.03%		99.61%	
Lack of Fit		<0.001 ***		<0.001 ***		<0.001 ***		<0.001 ***		<0.001 ***		<0.001 ***

*a*: dimensionless scaling coefficient; *k*: drying rate constant; *n*: empirical exponent; *b*: linear term coefficient; *D*_eff_: effective moisture diffusivity; *h*_m_: convective mass transfer coefficient. *** *p* < 0.001; ** *p* < 0.01; * *p* < 0.05.

**Table 3:** Analysis of variance (ANOVA) and regression coefficients of the predictive quadratic models for the techno-functional properties of dehydrated avocado seed flours.

Model Parameters	Techno-Functional Properties							
	SWC	(*p*-Value)	ORC		WRC		SOL	
Linear coefficient	5.036	<0.001 ***	2.017	<0.001 ***	10.849	<0.001 ***	7.448	<0.001 ***
X_1_ (Temperature—°C)	0.253	0.003 **	−0.007	0.823	0.149	0.089	0.027	0.908
X_2_ (Thickness—mm)	−0.002	0.976	0.021	0.530	0.192	0.031 *	0.286	0.221
X_3_ (Air velocity—m∙s^−1^)	0.001	0.991	−0.048	0.152	0.202	0.023 *	0.052	0.822
X_1_^2^	−0.119	0.313	0.168	0.001 **	−0.283	0.030 *	3.867	<0.001 ***
X_2_^2^	0.029	0.804	−0.058	0.239	−0.511	<0.001 ***	−1.410	<0.001 ***
X_3_^2^	−0.037	0.754	0.025	0.612	0.327	0.013 *	1.570	<0.001 ***
X_1_X_2_	−0.095	0.402	0.088	0.066	−0.006	0.962	−0.393	0.234
X_1_X_3_	−0.006	0.958	0.035	0.451	0.231	0.063	0.104	0.750
X_2_X_3_	−0.093	0.410	0.028	0.557	0.078	0.519	0.113	0.730
Model		0.213		0.031 *		<0.001 ***		<0.001 ***
Linear		0.028 *		0.468		0.008 **		0.659
Quadratic		0.748		0.007 **		<0.001 ***		<0.001 ***
Interaction		0.704		0.228		0.269		0.642
*R* ^2^	0.269		0.381		0.577		0.835	
Adj *R*^2^	0.081		0.221		0.468		0.793	
Predicted *R*^2^	0.000		0.000		0.289		0.724	
Lack of Fit (*p*-value)		0.015 *		<0.001 ***		0.055		0.015 *

SWC: swelling capacity; ORC: oil retention capacity; WRC: water retention capacity; SOL: solubility. Statistical significance is indicated by superscripts: *** *p* < 0.001; ** *p* < 0.01; * *p* < 0.05.

**Table 4:** Analysis of variance (ANOVA) and regression coefficients of the predictive quadratic models for the colorimetric properties of dehydrated avocado seed flours.

Model Parameters	Colorimetric Assessment							
	C*	(*p*-Value)	*h*°		BI		Δ*E*	
Linear coefficient	28.113	<0.001 ***	67.739	<0.001 ***	102.830	<0.001 ***	2.629	<0.001 ***
X_1_ (Temperature—°C)	0.195	0.214	0.129	0.441	1.130	0.002 **	−0.029	0.822
X_2_ (Thickness—mm)	0.172	0.271	−0.039	0.817	0.259	0.449	−0.056	0.662
X_3_ (Air velocity—m∙s^−1^)	0.600	<0.001 ***	0.126	0.453	2.120	<0.001 ***	−0.104	0.417
X_1_^2^	−0.376	0.106	−0.405	0.107	−1.364	0.010 **	0.213	0.262
X_2_^2^	−0.312	0.177	−0.069	0.780	−1.457	0.006 **	−0.054	0.773
X_3_^2^	−0.494	0.036 *	−0.631	0.014 *	−1.903	<0.001 ***	0.139	0.460
X_1_X_2_	0.174	0.429	−0.144	0.542	−0.129	0.789	−0.221	0.225
X_1_X_3_	−0.379	0.090	−0.670	0.007 **	−0.478	0.326	0.033	0.854
X_2_X_3_	0.399	0.075	−0.161	0.496	0.606	0.214	−0.300	0.102
Model		0.002 **		0.055		<0.001 ***		0.617
Linear		0.002 **		0.745		<0.001 ***		0.820
Quadratic		0.058		0.047 *		<0.001 ***		0.594
Interaction		0.089		0.043 *		0.457		0.243
*R* ^2^	0.488		0.352		0.696		0.171	
Adj *R*^2^	0.356		0.186		0.618		0.000	
Predicted *R*^2^	0.108		0.005		0.473		0.000	
Lack of Fit (*p*-value)		<0.001 ***		<0.001 ***		<0.001 ***		0.244

C*: Chroma; *h*°: Hue angle; BI: Browning Index; Δ*E*: Total color difference. Δ*E* was calculated using the freeze-dried avocado seed paste as the control reference (L* = 45.92, a* = 12.00, b* = 24.98). Statistical significance is indicated by superscripts: *** *p* < 0.001; ** *p* < 0.01; * *p* < 0.05.

**Table 5:** Analysis of variance (ANOVA) and regression coefficients of the predictive quadratic models for the bioactive properties of dehydrated avocado seed flours.

Model Parameters	Phenolic Content and Antioxidant Activity									
	TPC	(*p*-Value)	DPPH		ABTS		CHA		Ferulic Acid	
Linear coefficient	3.751	<0.001 ***	5.598	<0.001 ***	6.433	<0.001 ***	50.028	<0.001 ***	24.647	<0.001 ***
X_1_ (Temperature—°C)	−0.304	<0.001 ***	−0.379	<0.001 ***	−0.931	<0.001 ***	1.099	0.519	0.186	0.515
X_2_ (Thickness—mm)	−0.521	<0.001 ***	−0.400	<0.001 ***	−0.561	<0.001 ***	−5.092	0.005 **	−0.954	0.002 **
X_3_ (Air velocity—m∙s^−1^)	0.391	<0.001 ***	0.573	<0.001 ***	0.724	<0.001 ***	5.602	0.002 **	0.899	0.003 **
X_1_^2^	−0.526	<0.001 ***	−0.724	<0.001 ***	−0.588	<0.001 ***	−1.123	0.654	−1.396	0.002 **
X_2_^2^	0.266	<0.001 ***	0.409	<0.001 ***	0.093	0.510	−3.442	0.175	−0.923	0.033 *
X_3_^2^	0.149	0.011 *	0.016	0.885	1.050	<0.001 ***	−9.422	<0.001 ***	−1.631	<0.001 ***
X_1_X_2_	0.143	0.011 *	0.048	0.657	1.056	<0.001 ***	−0.250	0.917	0.430	0.290
X_1_X_3_	−0.300	<0.001 ***	−0.126	0.250	0.164	0.229	−6.949	0.006 **	−1.404	0.001 **
X_2_X_3_	−0.061	0.257	−0.054	0.616	−0.125	0.355	−3.485	0.153	0.522	0.201
Model		<0.001 ***		<0.001 ***		<0.001 ***		<0.001 ***		<0.001 ***
Linear		<0.001 ***		<0.001 ***		<0.001 ***		<0.001 ***		<0.001 ***
Quadratic		<0.001 ***		<0.001 ***		<0.001 ***		0.004 **		<0.001 ***
Interaction		<0.001 ***		0.614		<0.001 ***		0.024 *		0.005 **
*R* ^2^	0.939		0.829		0.905		0.572		0.648	
Adj *R*^2^	0.924		0.785		0.881		0.462		0.558	
Predicted *R*^2^	0.916		0.712		0.844		0.247		0.387	
Lack of Fit (*p*-value)		0.136		<0.001 ***		<0.001 ***		<0.001 ***		<0.001 ***

TPC: total phenolic content; DPPH: 2,2-diphenyl-1-picrylhydrazyl; ABTS: 2,2′-azino-bis(3-ethylbenzothiazoline-6-sulfonic acid); CHA: chlorogenic acid. Statistical significance is indicated by superscripts: *** *p* < 0.001; ** *p* < 0.01; * *p* < 0.05.

**Table 6:** Multi-response optimization scenarios and predicted values for dried avocado seed properties.

Parameter/Variable	Units	Scenario 1: Phytochemical Extract	Scenario 2: Solubility Profile	Scenario 3: Hydration Matrix
Operational Factors				
Temperature	°C	45.0	75.0	75.0
Thickness	mm	3.0	8.7	7.4
Air velocity	m s^−1^	2.5	0.5	0.5
Optimization Goal				
TPC	mg GAE g^−1^	Max		
CHA	μg g^−1^	Max		
Ferulic acid	μg g^−1^	Max		
SOL	%		Max	
SWC	mL g^−1^		Max	
WRC	mL g^−1^			Max
ORC	mL g^−1^			Max
Composite Desirability (*D*)		0.79	0.72	0.62
Predicted responses				
Drying time (*MR* = 0.05)	min	128.46	338.89	255.37
*D*_eff_ (×10^9^)	m^2^ s^−1^	4.63	14.67	15.69
BI		98.58	97.45	98.53
*h*°		67.36	67.30	67.35
TPC	mg GAE g^−1^	5.36	2.91	2.89
DPPH	mg TE g^−1^	6.88	4.13	4.01
ABTS	mg TE g^−1^	10.22	5.71	5.38
CHA	μg g^−1^	55.82	37.48	40.36
Ferulic acid	μg g^−1^	23.68	20.63	21.64
SWC	mL g^−1^	4.66	5.16	5.14
ORC	mL g^−1^	2.12	2.24	2.24
WRC	mL g^−1^	9.93	10.29	10.55
SOL	%	10.60	11.42	12.36

*D*_eff_: effective moisture diffusivity; BI: browning index; TPC: total phenolic content; CHA: chlorogenic acid; SWC: swelling capacity; ORC: oil retention capacity; WRC: water retention capacity; SOL: solubility. Note: In all scenarios, BI was minimized, and *h*° was maximized (for color preservation) to ensure product quality.

## Data Availability

The raw data supporting the conclusions of this article will be made available by the authors on request.

## Supplementary Materials

The following supporting information can be downloaded at: https://www.mdpi.com/article/10.3390/foods15142438/s1. Table S1. Reduced response surface models obtained by backward elimination for the responses that exhibited significant lack of fit in the full second-order model. Table S2. Residual diagnostics for the full second-order response surface models fitted to the Box-Behnken design. Table S3. Internal cross-validation of the response surface models at the central point of the Box-Behnken design (60 °C, 6 mm, 1.5 m s^−1^), using the three central-point replicates AS13, AS14, and AS15. Table S4. Retention of bioactive compounds, antioxidant capacity, and techno-functional properties in convectively dried avocado seed flours relative to the freeze-dried reference sample (AS0). Figure S1. Residual diagnostics for the twelve full second-order response surface models. Q-Q plots (top rows within each block) compare ordered standardized residuals with theoretical normal quantiles; the red solid line represents the identity y = x. Residuals versus fitted values plots (bottom rows within each block) show the distribution of re-siduals across the range of fitted values; the red dashed line represents residuals equal to zero. Responses shown: TPC, DPPH, ABTS, CHA, ferulic acid, BI (browning index), SWC, ORC, WRC, SOL, hue angle, and chroma. The total color difference (ΔE) was excluded from the multivariate analyses due to the absence of significant terms and is not shown. Figure S2. Gallic acid calibration curve used for the Folin-Ciocalteu total phenolic content assay. Data are expressed as mean ± SD from three independent measurements (*n* = 3) at each concentration level (10 to 150 μg mL^−1^). The linear regression equation and the coefficient of determination (R^2^) are indicated in the legend. Figure S3. Pareto chart of standardized effects (|t-statistic|) for each response surface model. Bars are colored red when the standardized effect exceeds the critical t-value at α = 0.05 (t_crit_ = 2.03, indicated by the vertical dashed line) and light blue otherwise. T: drying temperature; H: sample thickness; V: air velocity. Superscript 2 denotes quadratic terms; two-letter combinations denote interaction terms (TH, TV, HV).
