# Supplementary material for: Convective Drying of Avocado Seeds: Mass Transfer Thermodynamics and Multi-Response Optimization of Functional and Phytochemical Properties
Source: Foods. 2026 Jul 9;15(14):2438. doi: 10.3390/foods15142438 (PMC13408583; doi:10.3390/foods15142438)
Supplement: Supplementary file 1 [file foods-15-02438-s001.zip › foods-4392838-supplementary.pdf]

Supplementary Materials

Convective Drying of Avocado Seeds: Mass Transfer Thermodynamics and Multi-Response Optimization of Functional and Phytochemical Properties

Mayra Deyanira Ramírez-Aguirre [1], Ricardo de Jesús Montiel-López [2], Tomás García-Cayuela [1], Viridiana Tejada-Ortigoza [2], Veronica Rodriguez-Martinez [1] and Luis Eduardo Garcia-Amezquita [2,*]

[1] Escuela de Ingeniería y Ciencias, Tecnologico de Monterrey, Ave. General Ramón Corona 2514, Zapopan 45138, Jalisco, Mexico; a01114369@tec.mx (M.D.R.-A.); tomasgc@tec.mx (T.G.-C.); veronica.rodriguezmz@tec.mx (V.R.-M.)
[2] Escuela de Ingeniería y Ciencias, Tecnologico de Monterrey, Ave. Eugenio Garza Sada 2501, Monterrey 64700, Nuevo Leon, Mexico; a01740943@tec.mx (R.d.J.M.-L.); viri.tejada@tec.mx (V.T.-O.)
* Correspondence: garcia.amezquita@tec.mx

Contents

Table S1. Reduced response surface models by backward elimination

Table S2. Residual diagnostics

Table S3. Internal cross-validation at the central point

Table S4. Retention of bioactive and techno-functional properties versus the freeze-dried reference (AS0)

Figure S1. Residual diagnostics for the response surface models

Figure S2. Gallic acid calibration curve

Figure S3. Pareto standardized effects for each response

Table S1. Reduced response surface models obtained by backward elimination for the responses that exhibited significant lack of fit in the full second-order model.

Response	Full R^2	Full R^2adj	Full R^2pred	Full LoF p	Red R^2	Red R^2adj	Red R^2pred	Red LoF p	Retained terms
SWC	0.269	0.081	0.000	0.009	0.214	0.195	0.146	0.103	T
ORC	0.381	0.221	0.000	<0.001	0.319	0.251	0.124	<0.001	T, T^2, H^2, TH
ΔE	0.171	−0.042	0.000	0.405	0.000	0.000	0.000	0.571	(intercept only)
Hue	0.352	0.186	0.005	<0.001	0.334	0.249	0.123	<0.001	T, V, T^2, V^2, TV
Chroma	0.488	0.356	0.108	<0.001	0.395	0.317	0.156	<0.001	T, V, V^2, TV, HV

SWC: swelling capacity; ORC: oil retention capacity; ΔE: total color difference; Hue: hue angle (h°); Chroma: chroma (C*). Full: full second-order model including linear, quadratic, and interaction terms. Red: reduced model retaining only terms with $p \leq 0.10$ after backward elimination. R^2: coefficient of determination; R^2_{adj}: adjusted coefficient of determination; R^2_{pred}: predicted coefficient of determination; LoF p: lack-of-fit p-value. Retained terms: T = temperature; H = sample thickness; V = air velocity; superscript ^{2} denotes quadratic terms; two-letter combinations denote interactions.

Table S2. Residual diagnostics for the full second-order response surface models fitted to the Box-Behnken design.

Response	R^2	R^2adj	Shapiro W	Shapiro p	BP stat	BP p	Normality	Homoscedasticity
TPC	0.939	0.924	0.904	0.001	48.87	<0.001	Violated	Violated
DPPH	0.829	0.785	0.944	0.030	14.44	0.108	Violated	OK
ABTS	0.905	0.881	0.951	0.055	8.33	0.501	OK	OK
CHA	0.572	0.462	0.969	0.273	21.44	0.011	OK	Violated
Ferulic	0.648	0.558	0.974	0.405	13.21	0.153	OK	OK
SWC	0.269	0.081	0.975	0.425	14.58	0.103	OK	OK
ORC	0.381	0.221	0.988	0.910	18.02	0.035	OK	Violated
WRC	0.577	0.468	0.971	0.314	6.72	0.667	OK	OK
SOL	0.835	0.793	0.975	0.424	5.02	0.832	OK	OK
BI	0.696	0.618	0.966	0.200	10.41	0.319	OK	OK
Hue	0.352	0.186	0.953	0.067	13.96	0.124	OK	OK
Chroma	0.488	0.356	0.990	0.966	10.66	0.300	OK	OK
ΔE	0.171	−0.042	0.956	0.084	13.01	0.162	OK	OK

TPC: total phenolic content; DPPH, ABTS: radical scavenging capacities; CHA: chlorogenic acid; Ferulic: ferulic acid; SWC: swelling capacity; ORC: oil retention capacity; WRC: water retention capacity; SOL: solubility; BI: browning index; Hue: hue angle; Chroma: chroma; ΔE: total color difference. R^2, R^2_{adj}: coefficient of determination and adjusted coefficient of determination. Shapiro W and Shapiro p: statistic and p-value of the Shapiro-Wilk test for normality of residuals. BP stat and BP p: statistic and p-value of the Breusch-Pagan test for homoscedasticity of residuals. Normality and Homoscedasticity flagged as Violated when the corresponding test yielded $p < 0.05$.

Table S3. Internal cross-validation of the response surface models at the central point of the Box-Behnken design (60 °C, 6 mm, 1.5 m s^{-1}), using the three central-point replicates AS13, AS14, and AS15.

Response	Predicted	Observed mean	Observed SD	% Deviation
TPC	3.504	3.751	0.340	−6.6
DPPH	5.260	5.598	0.333	−6.0
ABTS	7.695	6.433	0.490	19.6
CHA	43.25	50.03	3.35	−13.6
Ferulic	23.66	24.65	0.77	−4.0
SWC	5.134	5.036	0.469	2.0
ORC	2.559	2.017	0.079	26.9
WRC	11.38	10.85	0.381	4.9
SOL	13.69	7.45	1.08	83.8
BI	108.5	102.8	1.01	5.5
Hue	72.77	67.74	1.17	7.4
Chroma	29.80	28.11	0.414	6.0
ΔE	3.015	2.629	0.434	14.7
Drying time	426.4	283.3	15.2	50.5
Deff (×10^{-9})	8.29	8.92	0.00	−7.1
hm	0.0687	0.0642	0.000	7.0

Predicted: value predicted by the full second-order model using coefficients fitted from all 15 experimental runs. Observed mean and Observed SD: mean and standard deviation of the three central-point replicates. Percentage deviation = 100 × (Predicted − Observed mean) / Observed mean. Units: TPC in mg GAE g^{-1} db; DPPH and ABTS in mg TE g^{-1} db; CHA and Ferulic in µg g^{-1} db; SWC, ORC, and WRC in mL g^{-1} db; SOL in %; BI as index units; Hue in degrees; Chroma dimensionless; ΔE dimensionless; Drying time in min; D_{eff} in m^2 s^{-1}; h_m in m s^{-1}.

Table S4. Retention of bioactive compounds, antioxidant capacity, and techno-functional properties in convectively dried avocado seed flours relative to the freeze-dried reference sample (AS0).

Treatment	Conditions	TPC obs	TPC ret%	DPPH obs	DPPH ret%	ABTS obs	ABTS ret%	CHA obs	CHA ret%	Ferulic obs	Ferulic ret%	SWC obs	SWC ret%	ORC obs	ORC ret%	WRC obs	WRC ret%	SOL obs	SOL ret%
AS1	45 °C, 3 mm, 1.5 m s−1	4.36	101.6	6.30	93.5	8.57	60.1	39.39	78.8	21.66	100.6	4.58	95.3	1.98	69.6	9.56	148.1	8.30	79.2
AS2	75 °C, 3 mm, 1.5 m s−1	3.57	83.2	5.59	83.0	4.29	30.1	59.69	119.3	23.88	110.9	5.45	113.3	2.08	73.2	9.74	150.8	9.59	91.4
AS3	45 °C, 9 mm, 1.5 m s−1	3.13	73.0	4.88	72.4	5.47	38.4	31.73	63.4	19.91	92.5	4.63	96.2	2.00	70.3	10.38	160.8	11.01	105.0
AS4	75 °C, 9 mm, 1.5 m s−1	2.91	67.9	4.36	64.8	5.42	38.0	51.04	102.0	23.86	110.8	5.12	106.3	2.45	86.3	10.54	163.1	10.72	102.3
AS5	45 °C, 6 mm, 0.5 m s−1	2.99	69.8	4.35	64.6	6.69	46.9	30.78	61.5	20.43	94.9	4.49	93.3	2.46	86.4	10.65	164.9	13.18	125.7
AS6	75 °C, 6 mm, 0.5 m s−1	2.88	67.1	3.70	54.9	4.80	33.7	29.27	58.5	20.90	97.1	4.83	100.4	2.08	73.1	10.62	164.4	12.57	120.0
AS7	45 °C, 6 mm, 2.5 m s−1	4.47	104.3	6.33	94.0	8.66	60.7	63.60	127.2	25.15	116.8	4.94	102.6	2.27	79.9	10.70	165.7	12.99	123.9
AS8	75 °C, 6 mm, 2.5 m s−1	3.16	73.6	5.18	76.8	7.43	52.1	34.29	68.6	20.00	92.9	5.26	109.3	2.04	71.6	11.60	179.6	12.80	122.1
AS9	60 °C, 3 mm, 0.5 m s−1	4.33	101.1	5.82	86.4	7.78	54.6	38.04	76.1	23.24	107.9	5.09	105.7	2.11	74.1	10.62	164.4	8.02	76.5
AS10	60 °C, 9 mm, 0.5 m s−1	3.31	77.3	5.66	84.0	6.77	47.5	32.80	65.6	19.26	89.5	5.41	112.3	1.94	68.2	10.42	161.3	7.01	66.9
AS11	60 °C, 3 mm, 2.5 m s−1	5.14	119.9	6.50	96.4	8.63	60.5	48.50	97.0	23.88	110.9	4.84	100.5	1.97	69.4	10.75	166.5	7.98	76.1
AS12	60 °C, 9 mm, 2.5 m s−1	3.88	90.4	6.11	90.7	7.12	49.9	29.32	58.6	21.99	102.2	4.78	99.4	1.92	67.4	10.87	168.3	7.42	70.8
AS13	60 °C, 6 mm, 1.5 m s−1	3.69	86.0	5.32	79.0	6.23	43.7	53.89	107.8	24.97	116.0	5.36	111.3	2.02	71.0	10.70	165.7	7.41	70.7
AS14	60 °C, 6 mm, 1.5 m s−1	3.73	87.0	6.03	89.5	7.06	49.5	49.43	98.8	25.01	116.2	5.01	104.1	2.08	73.1	10.98	170.1	6.38	60.9

Table S4. Retention of bioactive compounds, antioxidant capacity, and techno-functional properties in convectively dried avocado seed flours relative to the freeze-dried reference sample (AS0).

Treatment	Conditions	TPC obs	TPC ret%	DPPH obs	DPPH ret%	ABTS obs	ABTS ret%	CHA obs	CHA ret%	Ferulic obs	Ferulic ret%	SWC obs	SWC ret%	ORC obs	ORC ret%	WRC obs	WRC ret%	SOL obs	SOL ret%
AS15	60 °C, 6 mm, 1.5 m s−1	3.84	89.5	5.44	80.7	6.01	42.2	46.76	93.5	23.96	111.3	4.74	98.4	1.95	68.7	10.86	168.1	8.55	81.6

AS1 to AS15: convectively dried treatments (see Table 1 of the main manuscript for processing conditions). AS0: freeze-dried reference. Retention % = 100 × (observed value in dried sample) / (value in AS0). Reference values for AS0: TPC = 4.288 mg GAE g^{-1} db; DPPH = 6.738 mg TE g^{-1} db; ABTS = 14.256 mg TE g^{-1} db; CHA = 50.02 μg g^{-1} db; Ferulic = 21.53 μg g^{-1} db; SWC = 4.813 mL g^{-1} db; ORC = 2.842 mL g^{-1} db; WRC = 6.459 mL g^{-1} db; SOL = 10.482 %. TPC: total phenolic content; CHA: chlorogenic acid; SWC: swelling capacity; ORC: oil retention capacity; WRC: water retention capacity; SOL: solubility. obs: observed value; ret%: retention percentage.

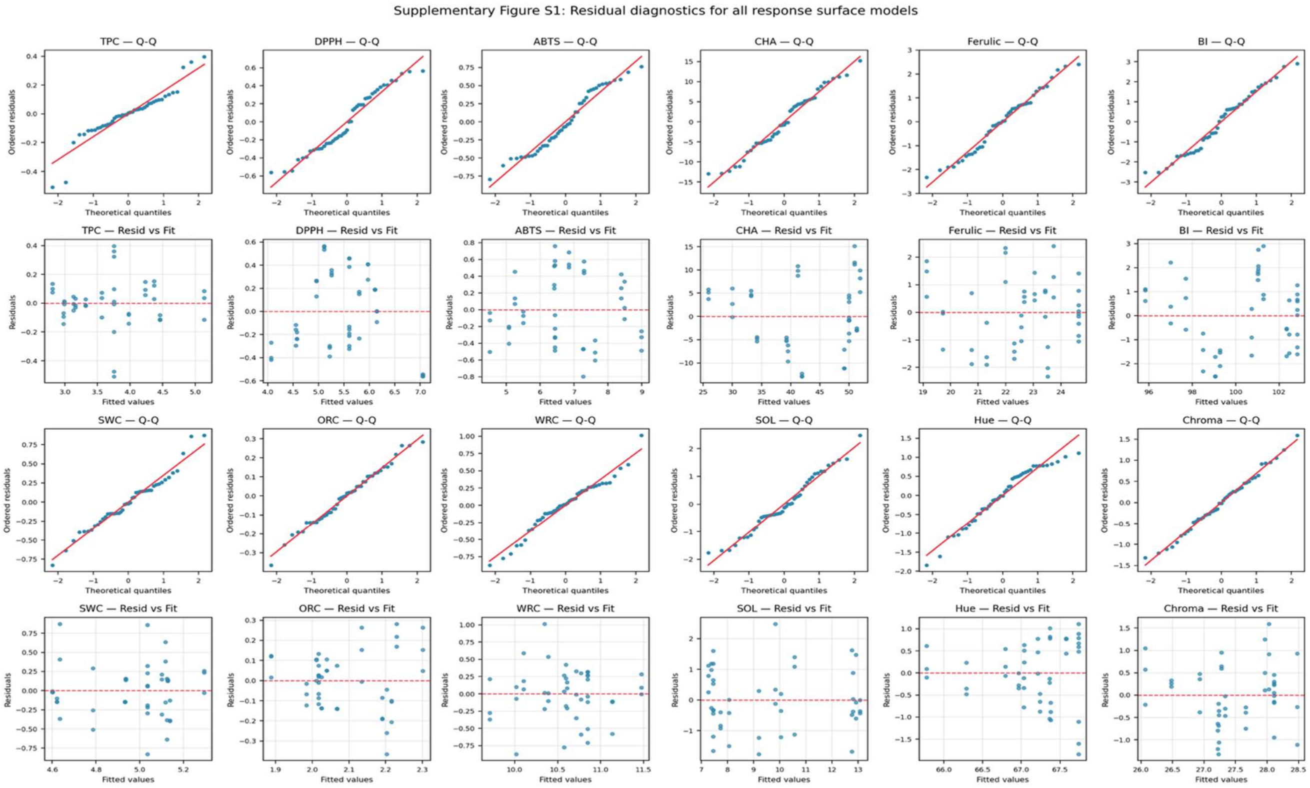

Figure S1. Residual diagnostics for the twelve full second-order response surface models. Q-Q plots (top rows within each block) compare ordered standardized residuals with theoretical normal quantiles; the red solid line represents the identity y = x. Residuals versus fitted values plots (bottom rows within each block) show the distribution of residuals across the range of fitted values; the red dashed line represents residuals equal to zero. Responses shown: TPC, DPPH, ABTS, CHA, ferulic acid, BI (browning index), SWC, ORC, WRC, SOL, hue angle, and chroma. The total color difference (ΔE) was excluded from the multivariate analyses due to the absence of significant terms and is not shown.

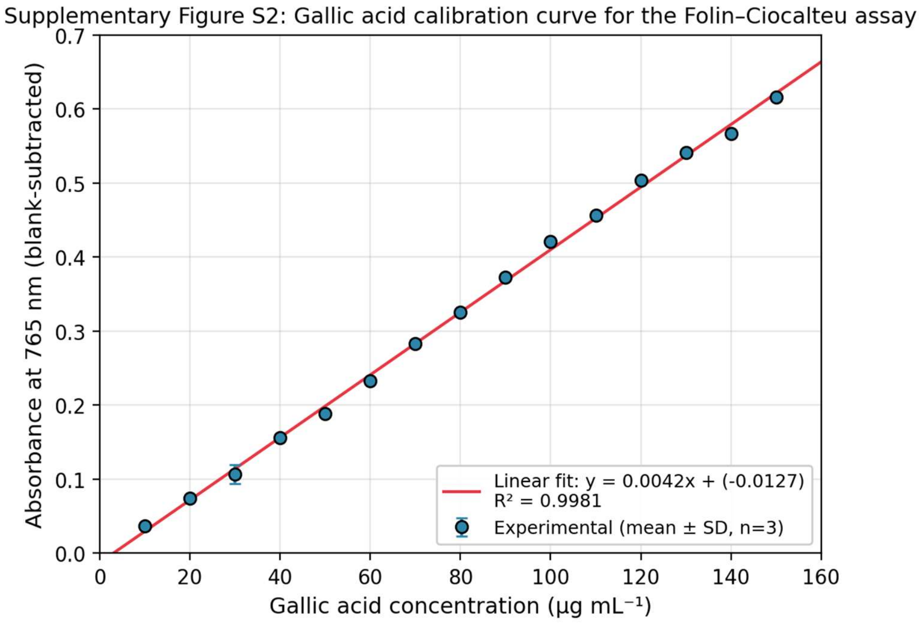

Figure S2. Gallic acid calibration curve used for the Folin-Ciocalteu total phenolic content assay. Data are expressed as mean ± SD from three independent measurements (n = 3) at each concentration level (10 to 150 μg mL^{-1}). The linear regression equation and the coefficient of determination (R^2) are indicated in the legend.

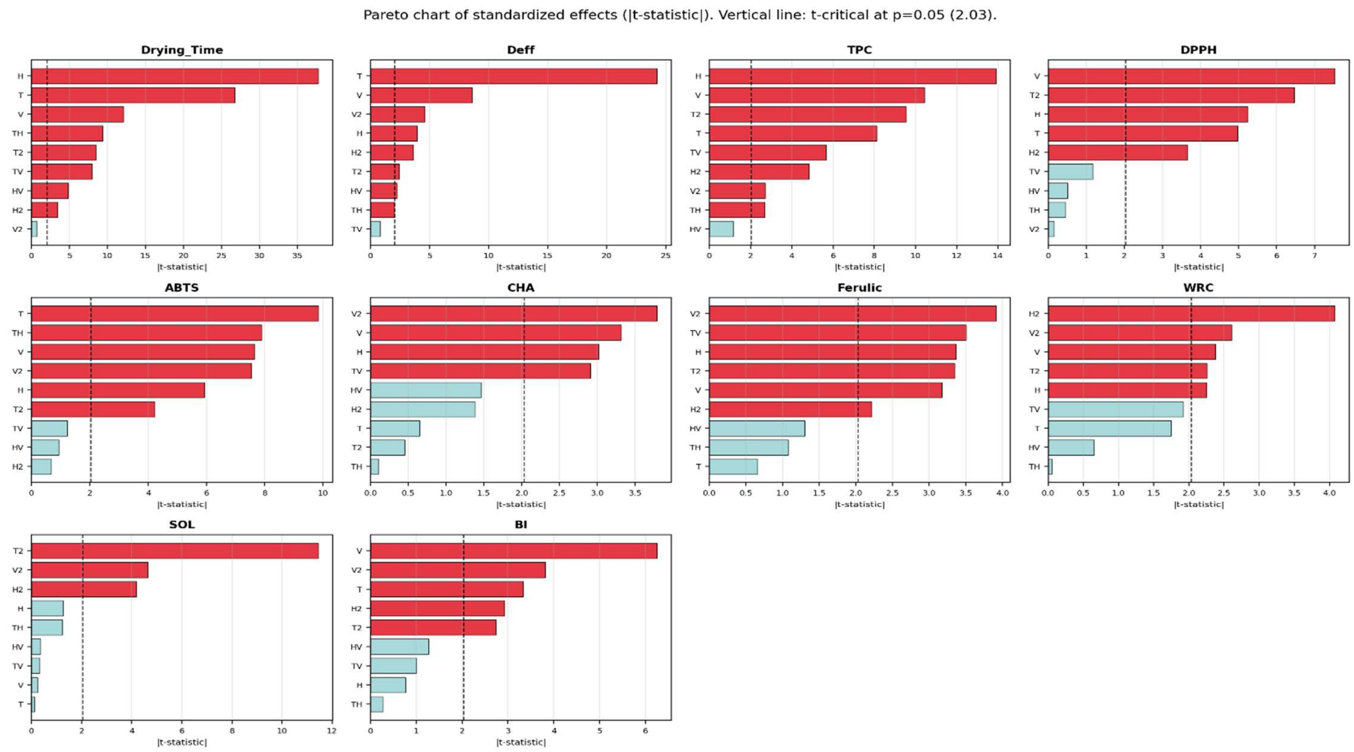

Figure S3. Pareto chart of standardized effects (|t-statistic|) for each response surface model. Bars are colored red when the standardized effect exceeds the critical t-value at α = 0.05 (t_{crit} = 2.03, indicated by the vertical dashed line) and light blue otherwise. T: drying temperature; H: sample thickness; V: air velocity. Superscript ^{2} denotes quadratic terms; two-letter combinations denote interaction terms (TH, TV, HV).
